# Evaluation of an event-driven 3FI ASIC for spectroscopic X-ray detection with synchrotron radiation

**DOI:** 10.1107/S1600577525010501

**Published:** 2026-01-01

**Authors:** Piotr Maj, Grzegorz W. Deptuch, Dominik S. Gorni, Giovanni Pinaroli, Gabriella A. Carini, David P. Siddons, Ryan Tappero, Soumyajit Mandal, Donald Pinelli, Timothy Kersten, Nicholas St John, Abdul K. Rumaiz, Anthony Kuczewski

**Affiliations:** a Instrumentation Department Brookhaven National Laboratory PO Box 5000 Upton NY11973-5000 USA; b NSLS-II Brookhaven National Laboratory PO Box 5000 Upton NY11973-5000 USA; Advanced Photon Source, USA

**Keywords:** synchrotron radiation, sparse data, pixel detector, fluorescence imaging, energy spectroscopy, front-end electronics, event-driven readout, low-noise measurements

## Abstract

The novel design and evaluation on the NSLS-II beamline of the 3FI application-specific integrated circuit (ASIC) bump-bonded to a simple, planar, 2D segmented silicon sensor are presented. The ASIC was developed for full-field fluorescence spectral X-ray imaging (3FI). It is a small-scale prototype that features a square array of 32 × 32 pixels, and the size of the pixels is 100 µm × 100 µm. The ASIC was implemented in a 65 nm CMOS integrated circuit fabrication process. Each pixel incorporates a charge-sensitive amplifier, a shaping filter, a discriminator, a peak detector and a sample-and-hold circuit, allowing detection of events and storage of signal amplitudes. The system operates in a frameless event-driven readout mode, outputting analog values for threshold-triggered events, allowing high-speed multi-element X-ray fluorescence data acquisition. The 3FI ASIC achieves per-channel spectrometric performance at a power consumption of only 200 µW per pixel, with nearly all dissipation confined to the analog front-end. An energy resolution is measured at the level of 308 eV full width at half-maximum (FWHM) at 8.04 keV (Cu *K*α), and 138 eV FWHM at 3.69 keV (Ca *K*α). This per-pixel capability makes the prototype suitable for *in situ* trace element microanalysis in biological and environmental studies. Moreover, the frameless architecture of the detector is designed to address limitations of conventional X-ray fluorescence microscopy, which typically requires mechanical scanning, by enabling continuous high-throughput data acquisition in future full-field implementations.

## Abstract 2

A new event-driven, energy-resolving ASIC intended for full-field X-ray fluorescence imaging is presented. Its frameless mode of operation was validated on a synchrotron beamline using a bump-bonded, 2D segmented silicon sensor, demonstrating per-pixel energy measurements with event-driven readout. Using on-chip spectral acquisition together with autonomous system control, this prototype establishes key building blocks for future real-time elemental mapping in biological, environmental and materials research.

## 1. Introduction

Hybrid pixel detectors have been widely used in X-ray imaging and scientific experiments for over 30 years. Their topology means that the choice of a sensor material is unconstrained by what the readout electronics need to be optimally fabricated on, making them particularly well suited for applications requiring high spatial resolution, wide dynamic range and good sensitivity. Most state-of-the-art detectors used at synchrotron facilities are hybrid pixelated detectors. Several influential systems have been pioneered at PSI, including Pilatus (Broennimann *et al.*, 2006 ▸), Eiger (Dinapoli *et al.*, 2011 ▸; Dinapoli *et al.*, 2013 ▸) and Jungfrau (Mozzanica *et al.*, 2018 ▸). Pilatus and Eiger were originally developed and deployed at the PSI beamlines, and consequently commercialized and further advanced by Dectris with new application-specific integrated circuit (ASIC) generations (Pilatus3 and Eiger2), as described by Donath *et al.* (2023 ▸) and Loeliger *et al.* (2012 ▸). At CERN, the Medipix and Timepix ASIC families have been developed (Ballabriga *et al.*, 2013 ▸; Yousef *et al.*, 2017 ▸), forming the basis for a wide range of detector systems subsequently realized by other institutes and companies. For example, the LAMBDA detector from DESY (Pennicard *et al.*, 2014 ▸) is based on Medipix3. Other notable developments include Rigaku’s XSPA (Zhang *et al.*, 2021 ▸) and the ePIX series from SLAC (van Driel *et al.*, 2020 ▸). Last but not least is the HEXITEC family from STFC (Veale *et al.*, 2018 ▸), which provides accurate spectroscopic energy information on a per-pixel basis but requires a pixel area of 250 µm × 250 µm, which is an order of magnitude larger than Eiger-, XSPA- or Medipix-based solutions, to achieve this capability. Its successor, HEXITEC MHz (Cline *et al.*, 2023 ▸), preserves spectroscopic performance while achieving dramatically higher throughput via MHz frame rates. Unlike event-driven systems, these detectors rely on very high readout speeds to handle count rates acceptably. Taken together, these detector families are optimized towards specific performance targets, including energy resolution, pixel size, maximum count rate and uniformity, where inter-pixel energy dispersion plays a crucial role.

**Figure 1:**
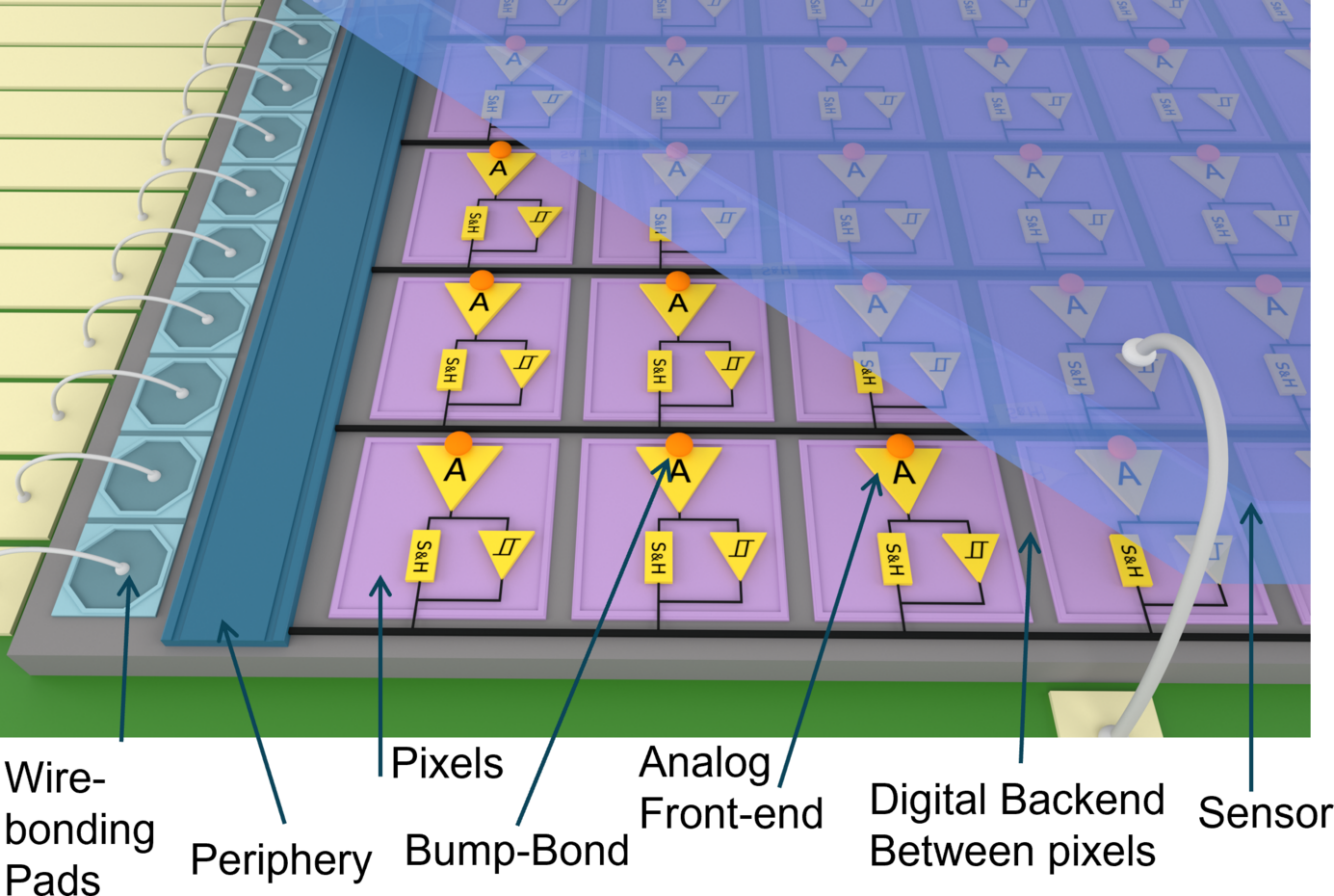
A hybrid pixel detector – idea of operation.

The operating principle of hybrid pixel detectors is illustrated in Fig. 1 ▸. A photon interacting with the sensor liberates charge in its active volume, producing a very short and low-amplitude current pulse that serves as the input to the readout electronics connected to the sensor with the bump-bonding technique. The readout integrated circuit (ROIC) ultimately defines the detector’s functionality and performance. Each pixel typically contains a charge-sensitive amplifier (CSA) and a pulse-shaping stage, which together determine noise characteristics and signal processing speed. Once the signal is shaped and filtered, it can, for example, be compared with a threshold and counted. This is the basis for the vast majority of commercial systems. More demanding applications require not only counting individual events but also measuring their amplitude and readout thereof without noticeable system latency. For more than two decades, researchers have been trying to enable per-pixel spectroscopy (Deptuch *et al.*, 2024 ▸), but limitations in technology nodes and ROIC backend architectures have hindered practical implementations. Most existing efforts can discriminate between a limited number of energy levels using multiple thresholds, but providing direct photon energy information usually yields a significant sacrifice of the achievable resolution.

Recent developments at Brookhaven National Laboratory (BNL) have led to a new generation of multi-channel, energy-discriminating readout ASICs, capable of capturing the amplitude of a pulse corresponding to each detected photon. This capability originates from a front-end analog architecture that employs pole-zero cancelation with a self-cascode field-effect transistor (SCFET)-based CSA (Deptuch & Otfinowski, 2025 ▸), which ensures precise amplitude measurement through low-noise operation and compensation of sensor leakage currents. In addition, a novel, in-house-developed event-based readout paradigm, EDWARD (Event-Driven With Access and Reset Decoder) (Gorni *et al.*, 2022 ▸), facilitates immediate output of digital address and analog signal values for each event, greatly easing data handling and throughput scaling. Such asynchronous and frameless readout architectures are particularly well suited for advanced X-ray imaging modalities, including X-ray fluorescence microscopy (XFM) and the emerging full-field fluorescence spectral X-ray imaging (3FI or FFFI).

### 1.1. Motivation for full-field X-ray fluorescence imaging

XFM is indispensable for mapping elemental compositions and distributions in biological, environmental and materials science samples (Copeland-Hardin *et al.*, 2023 ▸). Traditional scanning-based XFM methods, although capable of delivering excellent spatial resolution, suffer from long acquisition times because fluorescence data must be collected in steps of many points of exposure. This limitation inhibits efficient studies of dynamic biochemical processes, such as elemental transport, oxidation-state changes and chemical transformations in living systems (Kourousias *et al.*, 2020 ▸).

Full-field X-ray fluorescence imaging (3FI) addresses these challenges by simultaneously capturing fluorescence signals from an entire field of view, enabling near-real-time elemental imaging. In contrast to scanning systems, 3FI requires segmentation of a detector that can be achieved in large-area pixelated detectors (Romano *et al.*, 2014 ▸). This capability is especially valuable for studying biological interfaces such as plant–soil interactions, microbial redox processes and trace element dynamics situations, where elemental distributions and oxidation states can evolve rapidly (Brinza *et al.*, 2014 ▸).

In its targeted implementation, this detector, together with appropriate optics, is expected to provide elemental maps in 2D without mechanically scanning the sample, and 3D, elementally resolved tomograms with a single translation (Siddons *et al.*, 2020 ▸), which would significantly accelerate such experiments, particularly for biological samples. In this work, however, we focus on validating the underlying ASIC and readout concept rather than demonstrating full-field imaging performance.

### 1.2. Synchrotron-based evaluation of 3FI and paper structure

To advance towards full-field X-ray fluorescence imaging, the 3FI ASIC was designed, fabricated and hybridized with a sensor for spectrometric evaluation at NSLS-II. Its ability to capture trace element fluorescence spectra was assessed using standard fluorescent secondary radiation from reference samples.

This paper presents the design, implementation and experimental validation of a photon-spectrometric 3FI-based small-scale prototype detector. Section 2 introduces the architecture of the ASIC prototype. Section 3 describes the overall detector system and the integration strategy. Section 4 presents measurement results, focusing on the results achieved in experiments using a synchrotron beamline and highlighting the detector’s energy resolution and event-driven capabilities.

## 2. The 3FI ASIC for color X-ray imaging

The 3FI ASIC is suitable for reading out a pixelated semiconductor sensor. It was developed at BNL using a 65 nm CMOS process node, and it was specifically designed for near-real-time trace element microanalysis in complex biological systems.

**Figure 2:**
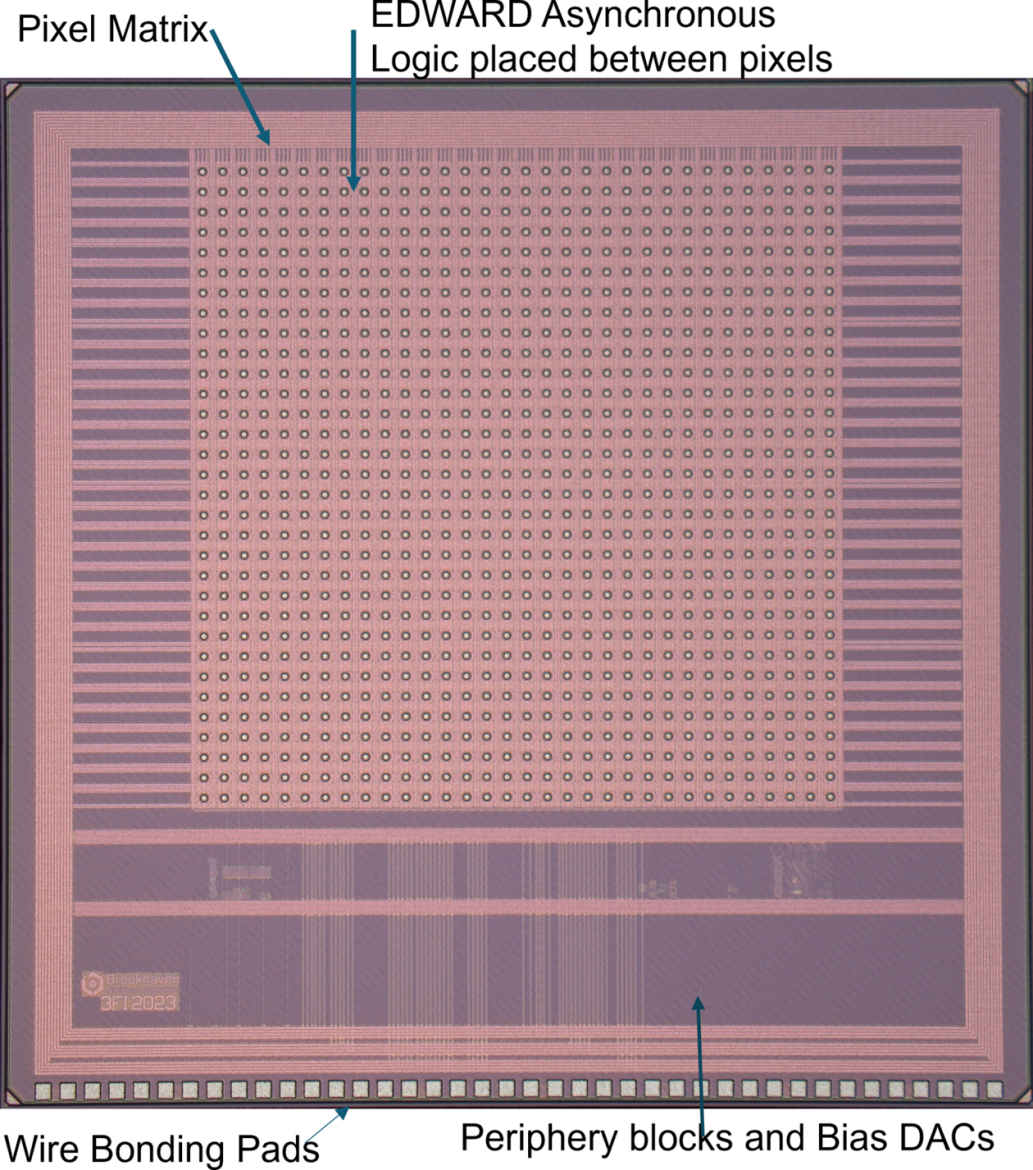
Photograph of the 3FI ASIC with visible wire-bonding pads placed on one side, being compatible with three-side buttable detector designs, periphery blocks for chip control, pixels and EDWARD logic layout are placed in between them.

A full-scale chip aims at a matrix of 256 × 256 pixels; however, the current prototype comprises an array of 32 × 32 square pixels. The pitch of a pixel is 100 µm. Each pixel in the pixel matrix works independently, having 24-bit memory keeping its current configuration. The operation of this matrix is supported by peripheral circuitry providing, among others, proper biasing, IC control and a temperature sensor. A photograph of the prototype chip is shown in Fig. 2 ▸.

**Figure 3:**
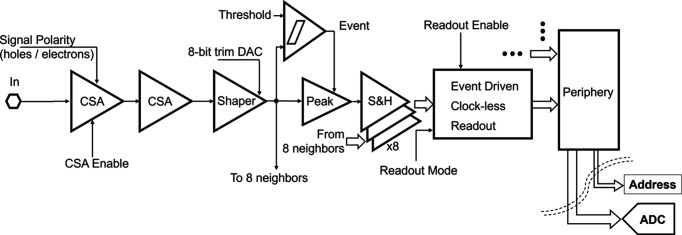
Simplified schematic diagram of a single channel in the 3FI ASIC.

A simplified block diagram of a single 3FI analog front-end channel is shown in Fig. 3 ▸. Each channel consists of a two-stage CSA with input signal polarity selection to work with holes or electrons as input signals, a third-order pulse-shaping filter, a discriminator with 8-bit offset correction, a peak detector and a bank of nine sample-and-hold (S&H) circuits. This configuration enables readout of the active pixel and its eight immediate neighbors. An active pixel is defined by the discriminator output, which triggers an event for the channel, and the system waits until a peak is achieved to latch the peak value and the eight neighbors. An event-driven asynchronous logic block coordinates the readout of the event address and its associated analog value. It transfers them via dedicated busses: the analog signal through the analog bus to the analog output buffer, and the digital address through the digital bus to the digital output buffer. A single channel contains a 24-bit memory, maintaining its individual configuration and enabling various functions, including signal polarity selection, charge amplifier and/or readout digital circuitry enabling/disabling, trimming digital-to-analog converter (DAC) value setting and readout mode selection.

Input charge processing is optimized for low-noise operation by employing a high-gain, two-stage amplifier followed by a third-order, semi-Gaussian pulse filter. When an input pulse exceeds the threshold level, the discriminator triggers a valid event, prompting the peak detector to capture the maximum signal value. This value is stored in the S&H array, which simultaneously latches the peak detector value of the central pixel and the shaper output values of its eight immediate neighbors. These latched values are then read out via the EDWARD asynchronous logic. Because the readout is strictly event-driven, the detector has no inherent frame rate. Performance is better expressed in terms of the event-acknowledge rate. Saturation occurs when the incident photon flux drives the aggregate event rate up to such service capacity. At this point, queues build up and additional events are increasingly rejected, but the system remains responsive (Gorni *et al.*, 2025 ▸).

**Figure 4:**
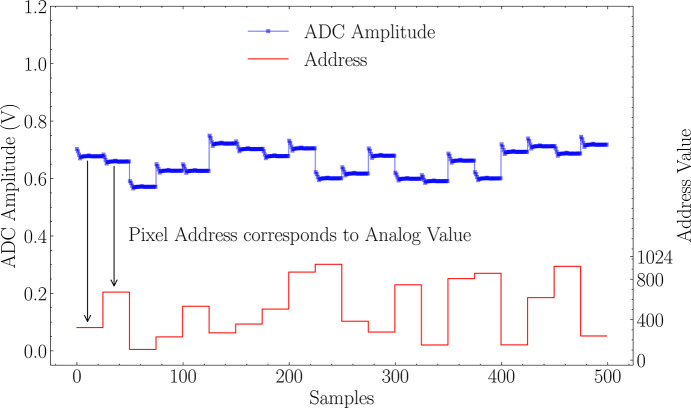
3FI readout modes: single-pixel readout mode, where the upper plot is assigned to the left scale, presenting the measured pulse amplitude in voltage, while the bottom plot presents the corresponding event address scaled to the right-side *Y* axis.

**Figure 5:**
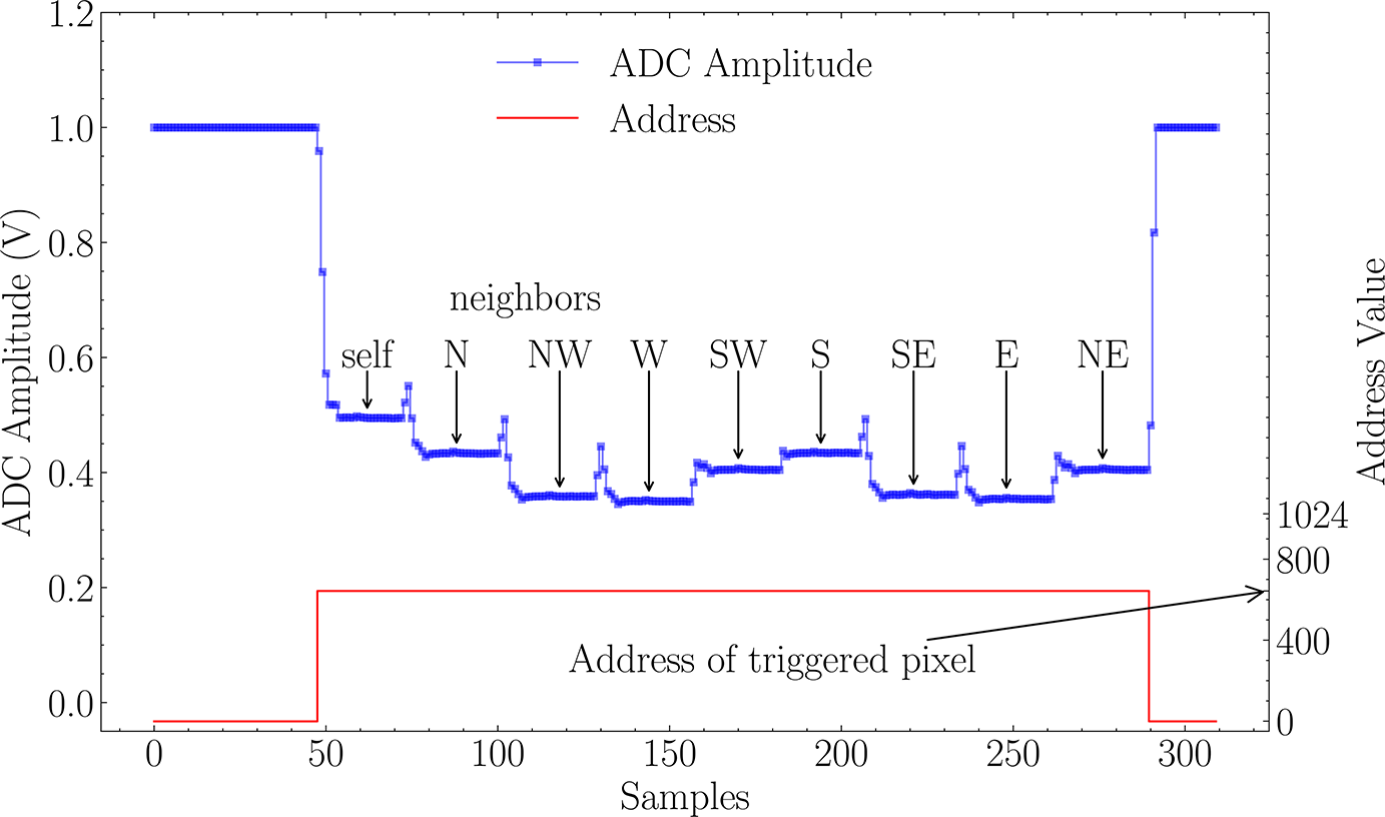
3FI readout modes: charge-sharing compensation readout mode, where the upper plot is assigned to the left scale, presenting the measured pulse amplitude in voltage, while the bottom plot presents the corresponding event address scaled to the right-side *Y* axis. The single event from the pixel address 648 triggers the readout of a central pixel together with all neighbors marked with geographic abbreviations (N, NE, …).

The 3FI ASIC outputs data through two channels. The first channel is for analog values. It features a differential analog output buffer that transmits the in-pixel-stored values of signal amplitudes. The second channel is digital, and it contains a high-speed serializer that outputs digital addresses of occurrences of radiation interaction events using the low-voltage differential signaling (LVDS)-compatible mode. The 3FI ASIC is designed for effectively zero-dead-time operation, meaning that the readout of an active event does not interfere with the registration of hits in the remaining channels. The maximum acknowledge rate of the digital readout is 18 MHz, corresponding to up to 18 million events per second per output link, or on average ∼17.6 k events per second per channel in a fully populated 1024-pixel configuration. At the same time, the ultimate per-channel event rate is constrained by the analog front-end recovery: with the peaking time of τ_p_ = 300 ns, approximately 7τ_p_ ≃ 2.1 µs is required for a complete return to the baseline, which sets the physical upper bound of about 500 kHz per channel. The 3FI ASIC can operate in two readout modes, as illustrated in Fig. 4 ▸ and Fig. 5 ▸. In both modes, all nine neighboring pixels are processed. In the single-pixel readout mode, the readout is limited to the central pixel, leaving the remaining eight values unread. In the second mode, referred to as the charge-sharing compensation readout mode, all nine neighboring pixels’ values are read out. Consequently, the reconstruction of the total photon energy can be achieved externally. While the single-pixel readout mode offers higher throughput, the charge-sharing compensation mode is designed to improve energy resolution by an off-chip correction.

## 3. The detector design

**Figure 6:**
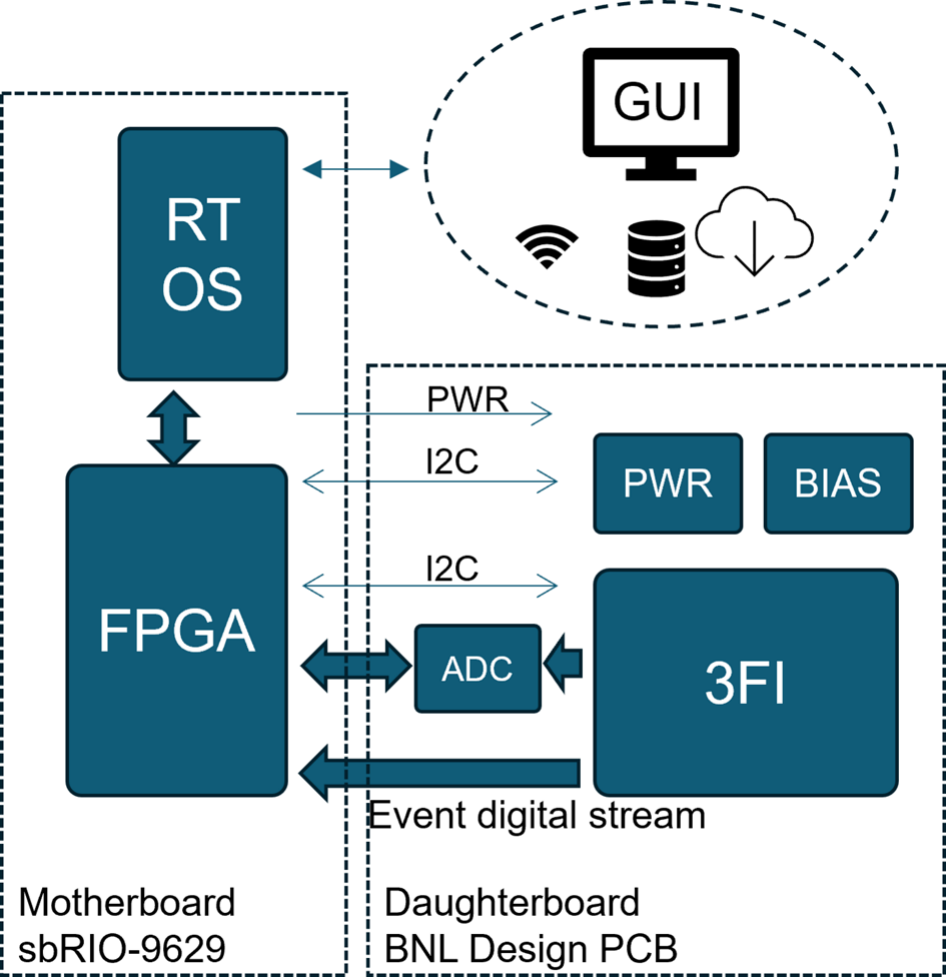
Simplified block diagram of 3FI-based detector.

To facilitate the evaluation of the 3FI ASIC, a fully integrated detector system was developed, supporting both autonomous and supervised operational modes. In the autonomous mode, the system can be controlled via TCP/IP commands, allowing for user-defined scripting and seamless integration with existing infrastructures, such as those available at the beamline. In the supervised mode, a graphical user interface (GUI) provides interactive control for configuration and monitoring purposes. The system is designed for versatility, featuring Peltier-based thermal stabilization to maintain optimal operating temperatures under varying environmental conditions. Additionally, it includes battery-powered and wireless communication capabilities, enhancing its portability and suitability for diverse deployment scenarios. A simplified block diagram of the detector is shown in Fig. 6 ▸. The system integrates two main components: an off-the-shelf high-performance embedded controller (sbRIO-9629) and a custom in-house-designed daughterboard. The sbRIO-9629 includes an Artix-7 FPGA and an Intel Atom E3845 quad-core 1.91 GHz processor, both fully programmable through software.

The presented approach for the architecture of the system results in minimal complexity of the daughterboard, focusing on the evaluation of the 3FI ASIC rather than assigning efforts to embedded system development. The daughterboard includes the 3FI ASIC bump-bonded to a 320 µm-thick silicon sensor, programmable voltage regulators, and both DAC and analog-to-digital converters (ADCs) for external biasing and monitoring. An AD9649 ADC [14-bit, up to 65 megasamples per second (MSps)] enables digitization of analog signals within the range of ±1 V.

The daughterboard interfaces with the embedded controller via a high-density connector, which carries both power and digital input–output signals. The FPGA is configured to perform the following functions:

(i) An I2C interface state machine with configurable clock and data lengths, used for slow control of the 3FI ASIC and auxiliary system components (*e.g.* power supply, DACs/ADCs).

(ii) High-speed transceivers with independently programmable regional clocks, bit-slip correction and latching delay, ensuring precise synchronization of the 14-bit digital data stream containing event pixel addresses.

(iii) Interface logic to the AD9649 ADC with reprogrammable sampling frequencies up to 65 MHz, automatic bit-slip correction and data delay tuning for accurate digitization of the analog waveform.

(iv) A digital stream filtering block detecting valid events by identifying the transition from synchronization patterns to actual event data, reducing FPGA–CPU data throughput.

The FPGA is managed by software running on a custom Linux real-time operating system (RTOS), which handles key tasks such as FPGA configuration and monitoring, pixel address and ADC data acquisition via direct memory access (DMA), data storage and an embedded GUI for interactive control. Ethernet connectivity (both wired and wireless) is supported for remote operation and integration with the synchrotron infrastructure.

**Figure 7:**
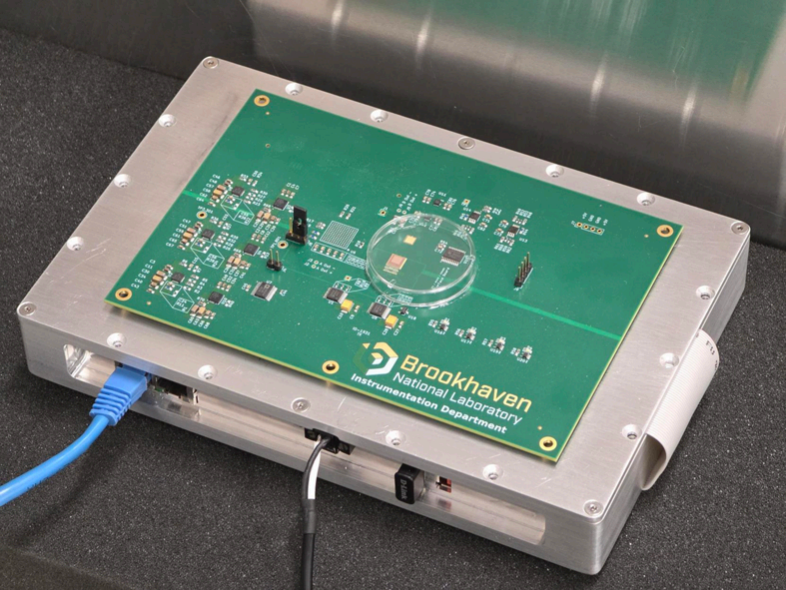
Photograph of the detector with the 3FI ROIC – before sensor assembly.

The sbRIO controller is housed within a metal enclosure that provides both passive heat dissipation and electromagnetic interference (EMI) shielding. A connector, placed on the enclosure, allows the 3FI daughterboard to be attached externally. A Peltier module is placed between the daughterboard and the enclosure to actively cool the sensor, maintaining temperatures below 0°C. A photograph of the complete detector is shown in Fig. 7 ▸.

The final system is a fully autonomous, single-photon energy-resolving detector that supports both local and remote operation. The present features include wireless connectivity, real-time data storage and live data preview. The detector was successfully deployed for its initial evaluation at the beamline 17-BM of NSLS-II [the X-ray Footprinting of Biological Materials beamline (XFP), https://www.bnl.gov/nsls2/beamlines/beamline.php?r=17-BM]. XFP delivers intense broadband pink beam (4.5–16 keV) X-rays from a three-pole wiggler source using a rhodium-coated toroid mirror to tailor beam size to experimental requirements via adjustable focusing of the toroidal mirror’s longitudinal radius and the distance to the sample from the source.

## 4. Measurements

**Figure 8:**
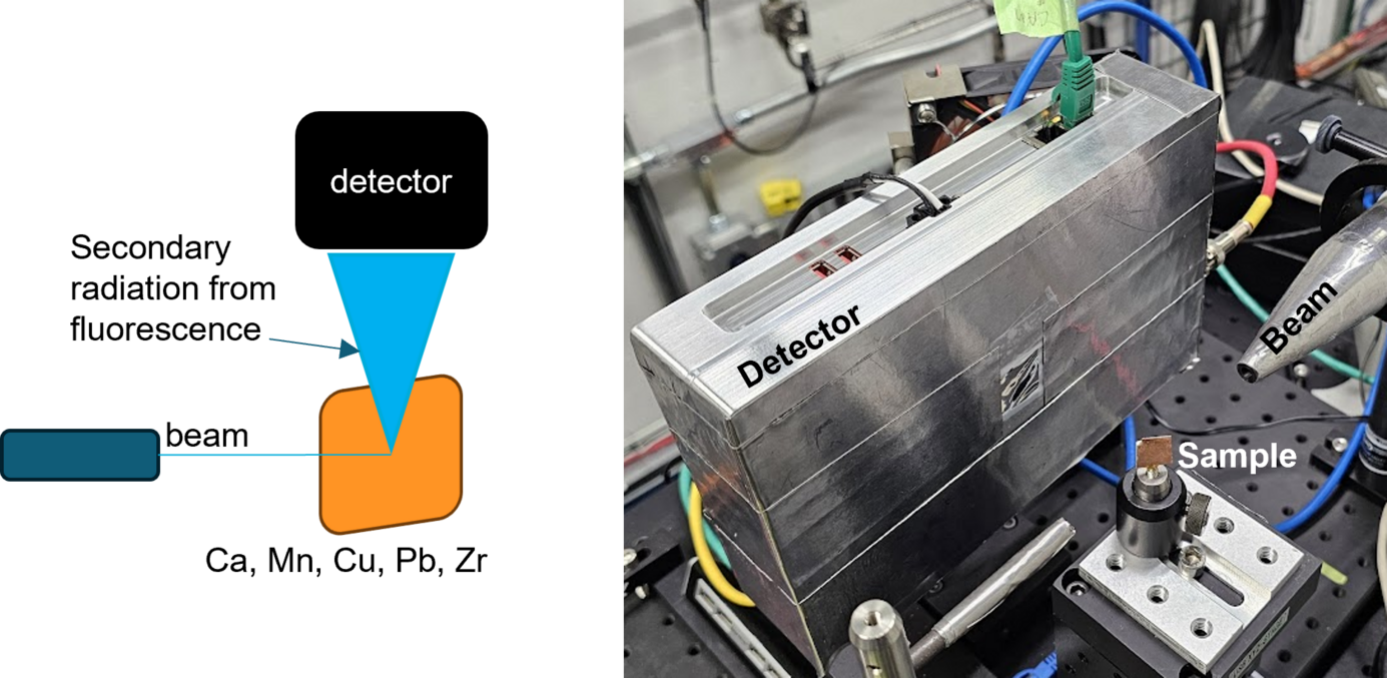
Measurement setup: schematic (left) and a setup photo (right) of fluorescence radiation from thin foils of Ca, Mn, Cu, Pb and Zr.

Comprehensive functional testing verified that both in-pixel and global configuration parameters precisely modulate the operational behavior of the 3FI ASIC, aligning with the intended design specifications. In particular, the ability to trim the voltage offset spread at the discriminator input was verified. Quantitative assessment of the 3FI’s spectrometric performance was carried out within the scenario conceptually shown in Fig. 8 ▸.

### 4.1. 3FI functionality evaluation

Validation of the detector commenced with a series of functional tests, defined here as those that confirm basic power-up, configuration, data-framing and event-handling behavior of the integrated system. The 3FI front-end ASIC dissipates ∼500 mW, including heat dissipation from the pixel array and periphery circuitry. Once the reference clock is present, it immediately transmits a synchronization word that establishes the sampling phase for the downstream FPGA. Correct bi-directional communication was verified through the I^2^C slow-control bus and through all dedicated control lines (supply rails, sensor-bias nodes and global threshold registers). A valid discriminator response, generated whenever an input pulse crosses the DC baseline, produces time-aligned digital addresses and analog samples that are captured without skew in the FPGA.

Although 3FI is inherently event-driven, the EDWARD provides a diagnostic ‘forced-read’ sequence that activates every pixel in turn (Gorni *et al.*, 2024 ▸). With the address stream and ADC samples thus aligned, a full-frame image of the 32 × 32 pixel plane was reconstructed, confirming channel ordering and analog-path integrity.

The detector’s maximum sustainable photon flux was adjusted to the acknowledge rate. In the present setup, the external readout chain reached 18 MHz. For the measurements reported here, we operated at the 8 MHz acknowledge rate, allowing the 40 MSps ADC to sample each latched analog value five times. Averaging these samples improved the per-event energy estimate while preserving the zero-dead-time architecture.

#### 4.1.1. Offset trimming

Following the initial verification of the proper 3FI’s responsivity, the next step was to trim the baseline offsets to enable efficient discrimination of pulse signals. One of the chip’s debugging modes enables readout of analog values from each pixel’s S&H circuit. By scanning through the full range of 8-bit trimming DAC settings, it is possible to match the baseline with the threshold level provided from the outside for each pixel.

**Figure 9:**
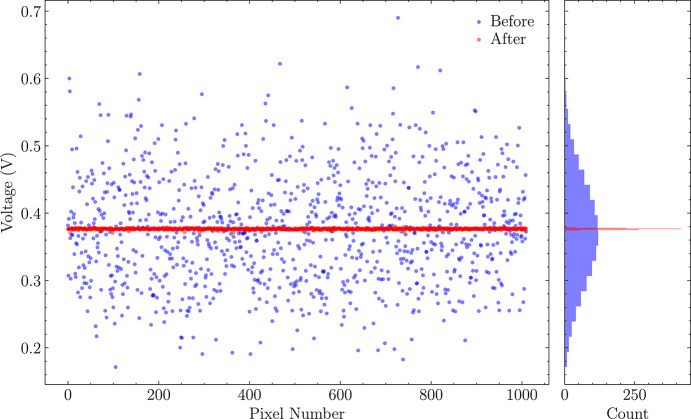
DC voltage measured with uniform trim DAC values (blue points) and after applying the trimming procedure (red points). Left: raw data; right: corresponding histogram.

Using these data, the optimal DAC settings for matching the baseline with the threshold level, provided externally, can be determined. This method provides a straightforward way to verify trimming performance. Results of this calibration are shown in Fig. 9 ▸. The standard deviation of DC levels for the same value placed inside the trimming DAC was 79 mV, and it was reduced to 0.98 mV after correction, which is consistent with the design assumptions and results of simulations.

In binary imaging systems (*e.g.* Eiger, Medipix), accurate adjustment of the threshold to approximately half of the deposited photon energy is critical for correct event detection (Zhang *et al.*, 2018 ▸). In contrast, in spectrometric systems such as the 3FI detector, the threshold serves primarily to trigger the peak detector, with the actual energy measurement performed afterwards. Therefore, the precision with which the threshold is set is less important.

To accelerate the trimming process, an alternative calibration method was developed, tailored to the requirements of event-driven readout architectures. In this approach, the global threshold is fixed at a target value, and all trim DACs are initially set to one extreme. This forces the pixel baselines to lie well below the threshold, resulting in a low rate of noise-triggered events. The trim DAC value for each pixel is then scanned towards the opposite extreme while the average event rate over a fixed time window is monitored. During the scan, each channel will cross the effective threshold at some point, producing a burst of events that subsequently diminishes as the trim DAC value increases further. The final trim DAC setting is defined as the value just beyond the threshold crossing, where noise-induced events are suppressed. This method enables efficient and scalable threshold equalization across the pixel matrix, which is completed in only a few seconds.

This iterative procedure is embedded in the detector control system and can be executed automatically, ensuring fast and reliable offset calibration whenever needed.

#### 4.1.2. Spectrometric performance

**Figure 10:**
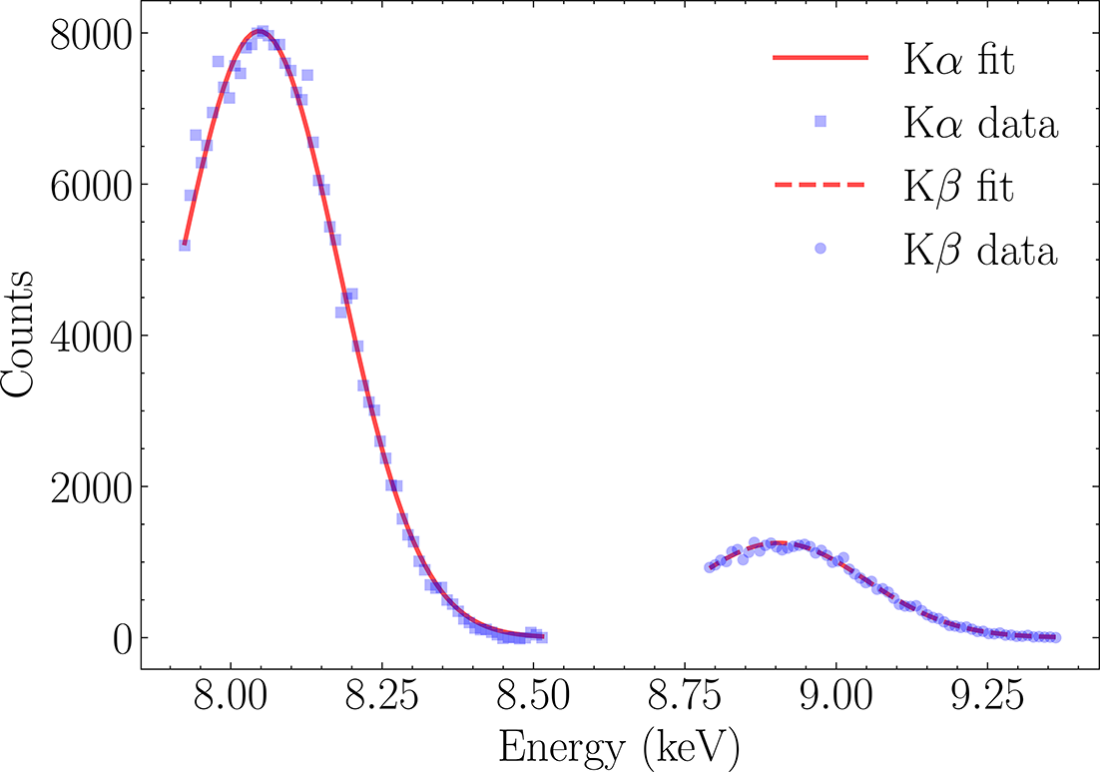
Fitting procedure for Cu *K*α and *K*β energy peaks.

The setup shown in Fig. 8  ▸ was employed to evaluate the spectrometric performance of the 3FI detector. Operating in the charge-sharing compensation mode, the single pixel latches its own pulse amplitude, simultaneously latching values from neighboring channels, enabling the characterization of both the baseline distribution in adjacent pixels and the energy-peak distribution in the active pixel. For the purposes of this paper, the analysis was done for a single pixel only. This decision was dictated by the unsatisfactory yield of the ASIC-sensor assembly. Energy resolution was assessed by acquiring fluorescence spectra from materials with well defined X-ray emission lines. Noise estimation was performed by fitting the right-hand side of each energy peak with a Gaussian function, as the left side typically contains background contributions (Bland, 1998 ▸). An example of this fitting procedure, applied to the Cu *K*α and *K*β lines, is presented in Fig. 10 ▸.

The target materials produced characteristic *K*α and *K*β fluorescence lines, with emission energies as follows:

(i) Calcium (Ca): *K*α at 3.691 keV, *K*β at 4.012 keV.

(ii) Manganese (Mn): *K*α at 5.898 keV, *K*β at 6.490 keV.

(iii) Copper (Cu): *K*α at 8.047 keV, *K*β at 8.905 keV.

(iv) Lead (Pb): *L*α at 10.551 keV, *L*β at 12.667 keV.

(v) Zirconium (Zr): *K*α at 15.775 keV, *K*β at 17.667 keV.

**Figure 11:**
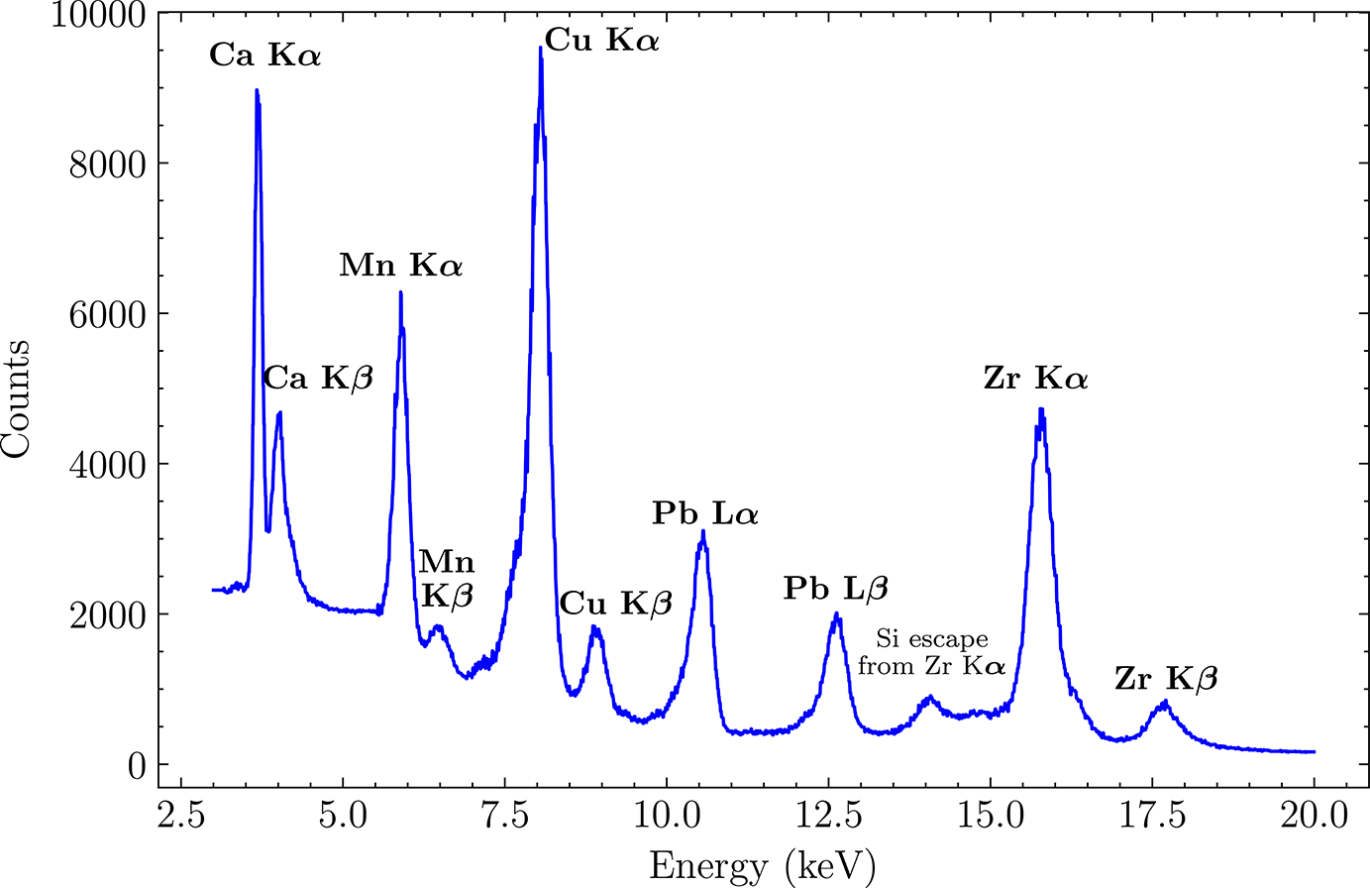
Combined energy spectrum from fluorescence targets.

The beam flux was adjusted in order not to exceed 10k events per single pixel per second, ensuring that the digital readout was not saturated, with the present acknowledge rate set to 8 MHz. To ensure statistical relevance, measurement durations were adjusted to collect more than 2 million events per target, resulting in a measuring time window on the order of hundreds of seconds. The spectra measured with different targets have different photon intensities. Therefore, they were normalized to match the background event rates and then combined into a single spectrogram, shown in Fig. 11 ▸.

The energy resolution for the Ca *K*α line was measured at 138 eV FWHM. The resolutions measured for other *K*α lines (and *L*α for Pb) were as follows: Mn at 249 eV, Cu at 308 eV, Pb at 339 eV and Zr at 469 eV. In all of the cases, clear α and β peak separation was observed, demonstrating low-noise per-pixel spectrometric performance of the prototype. No significant nonlinearity was observed in the evaluated energy range, indicating that the 3FI ASIC is compatible with color X-ray imaging concepts.

For each photon event detected in the central pixel, the readout also included values from adjacent pixels. These neighboring channels predominantly contained baseline values, as the probability of charge sharing with a particular neighboring pixel, especially corner pixels, was low. The distribution of these baseline measurements defines the baseline noise level, determined to be approximately 120 eV FWHM.

## 5. Conclusions

This work presents an initial experimental validation of the 3FI ASIC, featuring the EDWARD event-driven readout developed at BNL for energy-resolved X-ray fluorescence imaging. The 3FI ASIC features a 32 × 32 array of 100 µm × 100 µm pixels, each equipped with a CSA, shaping filter, peak detector and a bank of S&H circuitry. The ASIC operates in a frameless mode.

Integrated into a compact, fully autonomous detector system with embedded control, wireless connectivity and real-time data handling, the 3FI ROIC was successfully evaluated at the beamline 17-BM (XFP) at the NSLS-II. The detector demonstrated energy resolutions as good as 138 eV FWHM at 3.7 keV and 308 eV FWHM at 8.0 keV, with clear spectral separation across measured energy peaks. Neighboring pixels’ baseline noise distribution measured at 120 eV confirms the low-noise performance. Automated offset trimming and a low-noise front-end support stable operation and robust spectroscopic performance.

Designed with portability and integration in mind, the 3FI detector system can be readily deployed at synchrotron facilities. It interfaces directly with beamline control systems and is capable of controlling external hardware such as *XYZ* stages or other auxiliary instrumentation, supporting advanced experimental automation. In its current form, the system demonstrates key ASIC-level and system-level capabilities: single-photon energy resolution, frameless event-driven readout and autonomous operation that are essential for future full-field X-ray fluorescence imaging detectors. As such, the 3FI platform is a promising building block for next-generation instruments aimed at rapid, high-resolution elemental mapping of complex biological and environmental samples, although full-field imaging performance and benchmarking remain to be demonstrated.
